# Developing liposomal and ethosomal formulations with potent antioxidant and cytotoxic effects: unleashing the power of *Achillea millefolium* L.

**DOI:** 10.55730/1300-0144.6162

**Published:** 2025-11-13

**Authors:** Sena AKÇAKAYA MUTLU, Gökçe ŞEKER KARATOPRAK, Çiğdem YÜCEL, Selen İLGÜN, Esra KÖNGÜL ŞAFAK, Murat KOÇ, Esra KÜPELİ AKKOL

**Affiliations:** 1 Department of Pharmacognosy, Erciyes University Institute of Health Sciences, Erciyes University, Kayseri, Turkiye; 2 Department of Pharmacognosy, Faculty of Pharmacy, Erciyes University, Kayseri, Turkiye; 3 Department of Pharmaceutical Technology, Faculty of Pharmacy, Erciyes University, Kayseri, Turkiye; 4 Department of Pharmaceutical Botany, Faculty of Pharmacy, Erciyes University, Kayseri, Turkiye; 5 Department of Traditional, Complementary and Integrative Medicine, Public Health Institute, Ankara Yıldırım Beyazıt University, Ankara, Turkiye; 6 Department of Pharmacognosy, Faculty of Pharmacy, Gazi University, Ankara, Turkiye

**Keywords:** *Achillea millefolium*, ethosomes, liposomes, antioxidant, cytotoxicity

## Abstract

**Background/aim:**

*Achillea millefolium* L., a plant rich in phenolics and flavonoids, has been shown to possess strong antioxidant and cytotoxic properties. However, no pharmaceutical formulations have yet been developed for this plant. This study aimed to create and characterize an *A. millefolium* extract with high antioxidant and cytotoxic activities.

**Materials and methods:**

A total of 10 extracts were prepared from the plant’s aerial parts using ethanol concentrations ranging from 10% to 99.9%. The DPPH (1,1-diphenyl-2-picrylhydrazyl) and ABTS (2,2’-azino-bis(3-ethylbenzothiazoline-6-sulfonic acid)) radical scavenging tests were used to assess the antioxidant properties of the extracts. Cytotoxic effects were assessed using the MTT (3-(4,5-Dimethylthiazol-2-yl)-2,5-Diphenyltetrazolium Bromide) method on human colon cancer, cervical cancer, and breast cancer cell lines. The most active extract was further analyzed for its phenolic composition using high-performance liquid chromatography. This extract was then encapsulated in liposomes and ethosomes, known as nanocarrier systems, to enhance and prolong its efficacy.

**Results:**

The 99.9% ethanol extract exhibited strong DPPH scavenging activity with an IC_50_ value of 0.044 mg/mL and cytotoxic IC_50_ values ranging from 44.09 to 91.46 μg/mL across different cell lines.

**Conclusion:**

This study shows that absolute ethanol is a highly effective solvent for obtaining bioactive extracts from *A. millefolium*. Determining this is an important step in obtaining standardized extracts. In addition, the formulation studies conducted in this work confirm that the use of ethosomes and liposomes as nanocarriers for the formulation and delivery of this extract is a promising strategy.

## 1. Introduction

The family Asteraceae, which has up to 2945 genera and 33,867 species, is one of the richest groups of flowering plants in the world^1^. A member of this family, *Achillea* L., has a long history of use in traditional medicine [1]. In terms of ethnopharmacological history, *Achillea millefolium* L. is one of the most well-known species in the family. Known as “yarrow,” this plant has several traditional uses, including regulating menstruation, healing wounds, and stimulating appetite. It also acts as a diuretic and has carminative qualities [2]. In addition, an infusion made from its leaves reportedly has positive effects against cancer [3]. The following chemical elements of yarrow are considered bioactive: mono- and sesquiterpenoids, cyanidins, amino acids, salicylic acid, achillein (a bitter glycoside), lactones, flavonoids, tannins, coumarins, saponins, and sterols [4]. Considering the high secondary metabolite content of *A. millefolium* extracts, especially phenolic and flavonoid content, its bioavailability and stability are thought to be factors limiting the therapeutic effectiveness of these herbal products. The gastrointestinal system absorbs certain polyphenols, like flavonoids, but the plasma quantities are not high (1 μmol/L). The rapid metabolism of human tissues is the primary cause of this [5]. Different formulations at the nanoscale may be considered a prospective approach to enhance the bioavailability of *A. millefolium* extracts.

Their nanoscale size, high surface-to-volume ratio, and ability to modify physicochemical properties make nanopharmaceuticals superior to their bulk-phase equivalents [6]. Liposomes, the pioneers of nanoformulations that transcend solubility, bioavailability, and stability, show enormous potential as drug delivery vehicles owing to their hydrophobic and hydrophilic properties (and biocompatibility) [7]. Ethosomes, a distinct type of nanocarrier composed of phospholipids, water, and ethanol, were first introduced by Touitou et al. as an improved version of conventional liposomes [8,9]. Nanoformulations can be loaded with a wide range of natural and synthetic active ingredients, promising new medical and cosmetic treatments with improved efficacy, reduced side effects, and improved patient compliance.

This study tests the hypothesis that the therapeutic potential of traditionally used *A. millefolium* can be enhanced by incorporating it into modern nanopharmaceutical systems. To that end, the following experiments were conducted: the antioxidant activities of the extracts were evaluated using the Fe^+3^ to Fe^+2^ reducing capacity assay, as well as 1,1-diphenyl-2-picrylhydrazyl (DPPH^•^) and 2,2’-azino-bis(3-ethylbenzothiazoline-6-sulfonic acid) (ABTS^•+^) radical scavenging assays; cytotoxic activity was evaluated in COLO 205 (human colon cancer), HeLa (human cervical cancer), and MDA-MB-231 (human breast cancer) cell lines; the chemical composition of the absolute ethanol extract showing the greatest efficacy was analyzed using high-performance liquid chromatography (HPLC). Preparation, characterization, and release studies of liposome and ethosome formulations were also performed.

## 2. Material and methods

### 2.1. Plant material and extraction

The above-ground parts of *A. millefolium* were collected on 20 July 2022, at 11:00 AM from the Çubuk campus of Ankara Yıldırım Beyazıt University, and the herbarium specimen was catalogued at the Ankara Yıldırım Beyazıt University Public Health Institute Herbarium under the registration code Koç 3561.

The above-ground parts of the plant were firstly extracted three times using absolute, 90%, 80%, 70%, 60%, 50%, 40%, 30%, 20%, and 10% ethanol (Isolab, Eschau, Germany) on a magnetic stirrer at room temperature. Subsequently, the solvent was removed under reduced pressure using a rotary evaporator, and the remaining extracts were completely dried in a lyophilizer.

### 2.2. Assessment of total phenolic and flavonoid amounts

Using the Folin–Ciocalteu (Merck Chemicals, Darmstadt, Germany) method, the extracts’ total phenolic content was expressed as gallic acid (Sigma-Aldrich, Burlington, USA) equivalents [10]. Catechin (Sigma-Aldrich, Burlington, USA) equivalents were used to determine the extract’s total flavonoid content [11].

### 2.3. Determination of DPPH^•^ scavenging effect

The method of Hatano et al. was modified to evaluate the DPPH^•^ scavenging activity of the samples [12]. The extracts were dissolved at varying concentrations, and 100 μL of each extract solution was added to a 96-well plate, followed by 100 μL of DPPH^•^ solution (0.1 mM in ethanol) (Sigma-Aldrich, Burlington, USA). The absorbance at 517 nm was measured after 30 min of incubation in the dark at room temperature, and the percentage of radical scavenging activity was calculated. Chlorogenic acid (Sigma-Aldrich, Burlington, USA) was used as a standard. Sigma Plot 2001 (version7.0; SPSS Inc., Chicago, USA) was employed to calculate IC_50_ values through nonlinear regression curves.

### 2.4. Determination of ABTS^•+^ scavenging effect

Radical scavenging activity was assessed using Re et al.’s method [13]. An aqueous solution of ABTS (Sigma-Aldrich, Burlington, USA) and K_2_S_2_O_8_ (Merck Chemicals, Darmstadt, Germany) (2.45 mM, final concentration) was left in the dark for 12–16 h to get the ABTS^•+^ radical (7 mM), and the absorbance was adjusted to 0.700 (±0.030) at room temperature (734 nm). After mixing 900 μL of the radical solution with 100 μL of the extract solution, absorbance at 734 nm was recorded for 30 min at 1 min intervals. Trolox equivalent antioxidant capacity (TEAC) was used to calculate the percentage inhibition values.

### 2.5. Determination of reduction power

For this assay, 1 mL of the extract solution or standard chlorogenic acid was mixed with 2.5 mL of 0.2 M phosphate buffer (pH 6.6) (Merck Chemicals, Darmstadt, Germany) and 2.5 mL of 1% potassium hexacyanoferrate solution (Merck Chemicals, Darmstadt, Germany). This mixture was then incubated at 50 °C for 30 min, followed by the addition of 2.5 mL of 10% trichloroacetic acid (TCA) (Merck Chemicals, Darmstadt, Germany). Subsequently, the mixture was centrifuged for 10 min, and 2.5 mL of the resulting supernatant was mixed with 2.5 mL of water and 0.5 mL of 0.1% FeCl_3_ (Sigma-Aldrich, Burlington, USA). The reducing power of the extracts was expressed as ascorbic acid equivalents (AscAE) in mmol ascorbic acid per gram of sample, determined by measuring the absorbance of the mixture at 700 nm [14].

### 2.6. Cell culture

Cell culture studies were performed using COLO 205, HeLa, and MDA-MB-231 (ATCC, Manassas, USA) cell lines. Dulbecco’s Modified Eagle Medium (Capricorn Scientific, Ebsdorfergrund, Germany) supplemented with 10% fetal bovine serum (FBS) (Cegrogen Biotech, Stadtallendorf, Germany) and 1% penicillin (Corning, New York, USA) was used for the HeLa and MDA-MB-231 cell lines, whereas RPMI-1640 medium (Gibco; Life Technologies, Carlsbad, USA) supplemented with 10% FBS and 1% penicillin was used for COLO 205 cells.

### 2.7. Cytotoxic activity

COLO 205, HeLa, and MDA-MB-231 cells were counted and seeded in a 96-well microplate with 10,000 cells in 100 μL per well. At the end of 24 h, the medium was removed. After the extracts were dissolved in 0.1% DMSO, they were diluted with medium to obtain various concentrations (3.9–1000 μg/mL) and 100 μL of each sample was added to the wells as before. After 24 h, the wells were emptied and the MTT (Sigma-Aldrich, Burlington, USA) working solution, created by diluting the stock MTT solution in sterile phosphate-buffered saline (PBS) (Sartorius, Beit HaEmek, Israel), was dispensed into the wells of the plate. After 3 h, the medium was removed, and 100 μL of dimethyl sulfoxide (DMSO) (Merck Chemicals, Darmstadt, Germany) was added. After shaking for 5 min, the optical density of each well was measured at 570 nm using a microplate reader (Bio-Rad, Berkeley, USA), and cell viability (%) was calculated [15].

### 2.8. Analysis of phenolic compounds by high-performance liquid chromatography

A Shimadzu liquid chromatography system with a photodiode array (PDA) detector was employed to conduct HPLC analysis. A C18 analytical column measuring 250 × 4.6 mm with a particle diameter of 5 μm was used. The temperature was maintained at 22 °C throughout the analysis. The flow rate was adjusted to 1 mL/min. The mobile phase consisted of three components: A) methanol/water/acetic acid (10:88:2, v/v/v), B) methanol/water/acetic acid (90:8:2, v/v/v), and C) methanol. A gradient flow was used with varying proportions of these mobile phases over time. The retention times and UV spectra of the compounds separated by HPLC were determined by comparison with standards. The average values were reported after triplicate analysis of all standard and sample solutions. Methanol was purchased from Merck Chemicals (Darmstadt, Germany), and acetic acid was obtained from Sigma-Aldrich (Burlington, USA).

### 2.9. Formulation studies

#### 2.9.1. Preparation of liposomes

The liposome formulation was prepared using the Bangham thin-film hydration method. Dipalmitoyl phosphatidylcholine (Sigma-Aldrich, Burlington, USA) and cholesterol (Sigma-Aldrich, Burlington, USA) were dissolved together at a 1:1 molar concentration in a mixture of chloroform (Sigma-Aldrich, Burlington, USA) and methanol (Merck Chemicals, Darmstadt, Germany) (3:1 v/v). Subsequently, the organic solvents were evaporated at 42–44 °C, resulting in the formation of a dry lipid film. This film was then hydrated with *A. millefolium* absolute extract, selected for its antioxidant and cytotoxic properties. After being vigorously vortexed for 10 min, the mixture underwent a 10 min treatment in an ultrasonic bath to complete the liposome formation process, yielding a liposome suspension. The liposome suspension was centrifuged at 15,000 rpm for 30 min at 4 °C to separate the upper clear portion from the liposomes [16].

#### 2.9.2. Preparation of ethosomes

Ethosomes were prepared by the mechanical dispersion process. A mixture of soy phosphatidylcholine (Sigma-Aldrich, Burlington, USA), chloroform (Sigma-Aldrich, Burlington, USA), and methanol (Merck Chemicals, Darmstadt, Germany) (3:1 v/v) was prepared. The solvent was evaporated at 43 °C to obtain a dry lipid film. This film was hydrated with *A. millefolium* extract dissolved in 30% ethanol (Isolab, Germany), then vortexed for 15 min, and sonicated for 30 min. The resulting ethosomal suspension was then centrifuged at 10,000 rpm for 30 min to separate the upper clear portion from the precipitated ethosomes [17].

#### 2.9.3. Characterization of formulations

##### 2.9.3.1. Determination of particle size, distribution, and measurement of zeta potential values

Zetasizer Nano ZS (Malvern Panalytical, Malvern, UK) was used to measure particle size, size distribution, and zeta potential. The measurements were performed in six parallel runs.

##### 2.9.3.2. Loading efficiency

Formulations were centrifuged, and the loading efficiency (LE%) was calculated from the supernatant using the above-mentioned HPLC method according to the following equation [16].


Equation LE%: Total extract amount - unloaded extrac amount

The total amount of extracts

##### 2.9.3.3. In vitro release studies

In vitro release experiments were conducted in phosphate-buffered saline (PBS) (pH 7.4; Oxoid Ltd., Altrincham, England) using Franz diffusion cells with a receptor volume of 2 mL. The dialysis membrane (MA~12,000 Da) was soaked in pH 7.4 phosphate buffer overnight and placed between the Franz cells. After adding 1 mL of the suspended formulations to be released to the upper half of the cells and 2 mL of PBS to the lower part, the ambient temperature was set at 37 °C and the mixing speed was maintained at 400 rpm. The amount of released extract was determined from samples collected at the end of 24 h [17].

### 2.10. Equipment used in the experiments

Rotavapor (Ilmvac vacuum pump; Ilmvac, Ilmenau, Germany), rotary evaporator (Laborota 4000 efficient; Heidolph, Schwabach, Germany), lyophilizer (Freezone 4.5; Labconco, Kansas City, USA) and magnetic stirrer (MR 3001; Heidolph, Schwabach, Germany) were used during the extract preparation phase.

An ultrasonic bath (S 100H Elmasonic; Elma Schmidbauer, Singen, Germany), water bath (Memmert, Büchenbach, Germany), spectrophotometer (UV-1800; Shimadzu, Kyoto, Japan), pH meter (pH 720 WTW; Inolab, Weilheim, Germany), and vortex mixer (ZX Classic; Velp Scientifica, Usmate, Italy) were used for antioxidant activity measurements.

Centrifuges (SL 16R; Thermo Fisher Scientific, Waltham, USA; and 320R; Hettich, Kirchlengern, Germany), an ultrasonic bath (S 100H Elmasonic; Elma Schmidbauer, Singen, Germany), a microplate reader (Synergy HT; BioTek Instruments, Vermont, USA), a sterile cabinet (Safe Fast Elite; Faster-Air, Cornaredo, Italy), a cell counter (Cedex XS; Roche Innovatis, Bielefeld, Germany), and a CO_2_ oven (Galaxy 170R; New Brunswick Scientific, Edison, USA) were used for cytotoxicity determination.

High-performance liquid chromatography (1200 Series; Agilent Technologies, Santa Clara, USA) was used for content determination.

Rotavapor (Labor Vacuum System; Ilmvac, Ilmenau, Germany; 4000 Efficient; Heidolph, Schwabach, Germany), and shaking water bath (ST 30; Nuve, Ankara, Türkiye) were used in the formulation stage.

### 2.11. Statistical analysis

Tukey’s and Dunnett’s post hoc tests were used to determine significant differences between means following one way ANOVA. A p < 0.05 was considered statistically significant.

## 3. Results and discussion

### 3.1. Assessment of total phenolic and flavonoid amounts

Upon examination of the phenolic content, ACM90 (90% ethanol extract) and ACM99.9 (absolute ethanol extract) extracts were found to have the highest content, with values of 146.24 ± 0.80 and 140.23 ± 16.51 mg_GAE_/g_extract_, respectively. The ACM10 (10% ethanol extract) showed the lowest total phenolic content, with a value of 83.30 ± 1.60 mg GAE/g extract. Similar results were determined in the total flavonoid content analysis, and the ACM90 and ACM99.9 extracts were found to have the highest content with values of 62.73 ± 1.26 and 58.31 ± 1.31 mg_CA_/g_extract_, respectively (Table 1). In the research conducted by Andleeb et al., the total phenolic content in the *A. millefolium*’s aerial parts extract made with 70% ethanol was measured at 123.87 ± 0.21 mg_GAE_/g_extract_. In our study, the ACM70 (70% ethanol extract) extract aligns closely with the findings of Andleeb et al., displaying a total phenolic content of 143.77 ± 2.13 mg_GAE_/g_extract_. In the same study, the total amount of flavonoids was defined as milligrams of quercetin per gram of extract made with 70% ethanol and was found to be 42.10 ± 0.29 mg_QE_/g_extract_[18]. In another research, the polyphenolic content of *A. millefolium*’s aerial parts methanolic extract was found to be 281.7 mg/g [19]. According to the study conducted by Keser et al., *A. millefolium* flower (ethanol, water), leaf (ethanol, water), and seed (ethanol, water) extracts yielded respective quantities of 74, 134, 78, 128, 70, and 126 mg/g DW of total phenolics expressed as quercetin equivalents (QETP) [20]. Although the results obtained from the studies are close to each other, the differences may be attributed to such factors as the difference in the standards used in the test method, the solvent of the extracts, the time and place of collection of the plant.

### 3.2. Antioxidant activity studies

The outcomes of the studies conducted on *A. millefolium* extracts showed that the extracts possessed potent DPPH scavenging abilities. According to Table 1, the ACM60 (60% ethanol extract), ACM70, ACM80 (80% ethanol extract), ACM90, and ACM99.9 extracts showed statistical significance, as evidenced by their IC_50_ values of 0.037–0.044 mg/mL (p > 0.05). The extract with the limited activity was ACM10 extract, which was also determined to have low content in total phenolic and flavonoid analysis. The scavenging effects of all extracts on ABTS^•+^ were assessed, and the results are outlined in Table 1. At a concentration of 0.5 mg/mL, TEAC values were measured at 2.29 ± 0.16 for ACM70, while at 0.25 mg/mL, a Trolox equivalent of 1.60 ± 0.12 mmol/L was detected. Both ACM10 and ACM20 extracts showed a statistically significant effect at both concentrations studied (p > 0.05). It was found that the lower polarity extracts ACM80, ACM90, and ACM99.9 showed strong activity, although they were not as active as ACM70. Candan et al. reported an IC_50_ value of 45.60 ± 1.30 μg/mL for DPPH radical scavenging activity in the chloroform (CHCl_3_) fraction of the methanol extract from the aerial parts of *A. millefolium* [2]. Lidiya et al. also used ABTS^•+^ and DPPH• assays to measure antioxidant capacity The DPPH experiment on plant water extracts yielded TEAC_DPPH_ values of 24.15 ± 0.15 to 116.74 ± 0.21 μM TE/g DW [21]. Similarly, TEAC_ABTS_ values were 18.59 ± 0.22 to 125.75 ± 2.24 μM TE/g DW.

**Figure 1:**
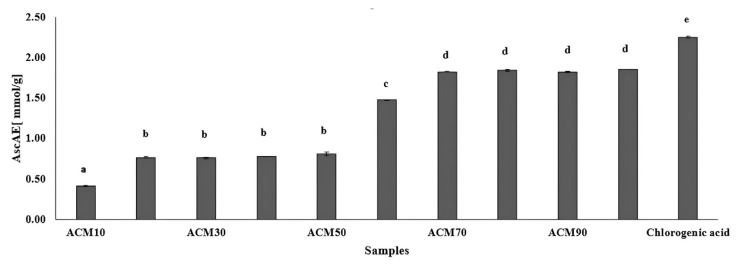
Reducing capacity of extracts and chlorogenic acid. Values labeled with identical letters (a–e) indicate no statistically significant differences between them (p > 0.05). ACM10: 10% ethanol extract; ACM20: 20% ethanol extract; ACM30: 30% ethanol extract; ACM40: 40% ethanol extract; ACM50: 50% ethanol extract; ACM60: 60% ethanol extract; ACM70: 70% ethanol extract; ACM80: 80% ethanol extract; ACM90: 90% ethanol extract; ACM99.9: absolute ethanol extract.

The conversion of iron (III) to iron (II) by the extracts was determined and expressed in terms of equivalent ascorbic acid (AscAE). The absolute ethanol extract showed the highest activity, with an AscAE value of 1.85 ± 0.002 mmol/g. However, the ACM60, ACM70, ACM80, and ACM90 extracts also showed significant activity (p > 0.05). The ACM10 extract had the lowest activity with an AscAE[mmol/g] value of 0.41 ± 0.006 (Figure 1). None of the extracts matched chlorogenic acid’s activity. Recent research indicated that extracts from the aerial parts of *A. millefolium*, prepared using choline chloride: lactic acid (1:2) deep eutectic solvent and 80% ethanol had Ferric Reducing Antioxidant Power (FRAP) values of 58.52 ± 0.34 and 22.44 ± 0.20 mg Fe^2+^ g^−1^ DW, respectively [22]. In another work by Lidiya et al., FRAP activity ranged from 29.57 ± 0.40 to 132.71 ± 1.86 μM TE/g ka [21]. Phenolic and flavonoid content in extracts and antioxidant activity are linked, suggesting a significant association.

### 3.3. Cytotoxic activity

The ACM90 and ACM99.9 extracts had the lowest IC_50_ values in the COLO 205 and HeLa cell lines. In the MDA-MB-231 cell line, it was determined that the ACM70 extract had the lowest IC_50_ with a value of 30.45 ± 5.15 μg/mL (Table 2). The ACM99.9 extract, with an IC_50_ value of 44.09 ± 1.41 μg/mL, displayed similar statistical significance to the ACM70 extract (p > 0.05). The IC_50_ values of the ACM99.9 and ACM90 extracts in the HeLa cell line were found to be 91.46 ± 3.00 and 97.01 ± 7.74 μg/mL, respectively. In the COLO 205 cell line, the extracts showing the highest activity were determined to be ACM90 and ACM99.9 extracts (Table 2). IC_50_ values for ACM90 and ACM99.9 in the COLO cell line were found to be 50.63 ± 3.12 and 52.68 ± 2.71 μg/mL, respectively. Considering the results of the MTT experiment performed on the MDA-MB-231 cell line, the most effective extract was ACM70. The IC_50_ value of this extract in the MDA-MB-231 cell line was calculated at 30.48 ± 5.15 μg/mL (Table 2). However, the ACM99.9 extract was found to be equally statistically significant. Amirghofran and Karimi found that *A. millefolium* 70% ethanol extract inhibited HeLa cells by less than 50% at doses of 10, 50, 100, and 400 μg/mL. In this study on cytotoxic action, *A. millefolium* extract in 70% ethanol reduced MDA-MB-435 cell viability by over 50% at 50, 100, and 400 μg/mL doses [23]. According to Pereira et al. [24], the hydroethanolic extract from *A. millefolium* had a GI_50_ of 70.8 μg/mL on HCT-15 human colorectal cells, limiting cell proliferation. This bioactivity may be attributable to the extract’s high phenolic acid content, notably chlorogenic acid derivatives, which make up 80% of it. In the antioxidant and cytotoxicity tests, the most effective extract was found to be the ACM99.9 extract, and the decision was made to proceed with this extract in the formulation studies.

### 3.4. HPLC analysis

**Figure 2:**
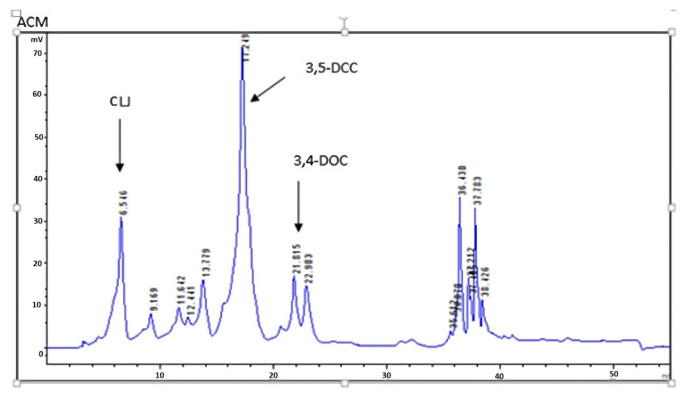
HPLC chromatogram of the ACM99.9 extract. **1**: Chlorogenic acid; **2:** 3,4-dicaffeoylquinic acid; **3:** 3,5-dicaffeoylquinic acid

**Figure 3:**
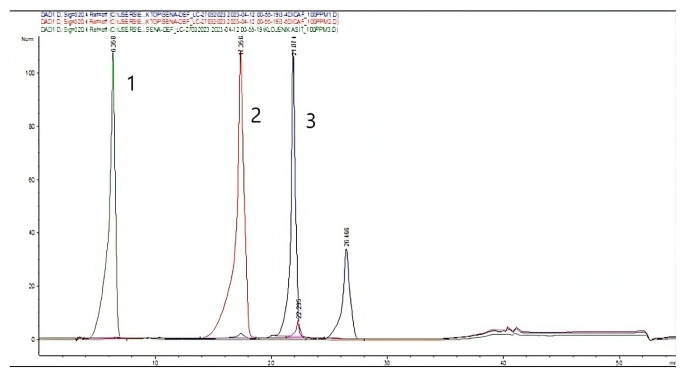
HPLC chromatogram of standards.

As the ACM99.9 extract was found to be the most effective extract as a result of antioxidant and cytotoxicity experiments, this extract continued to be studied in the formulation phase. The ACM99.9 extract selected for formulation was analyzed by HPLC. The HPLC chromatogram of the ACM99.9 extract given in Figure 2, the chromatogram of standards given in Figure 3, and the obtained results are given in Table 3. In the resulting chromatogram, the peak at minute 6.546 was defined as chlorogenic acid, the peak at minute 17.249 was defined as 3,5- dicaffeoylquinic acid (3,5-DCCA) and the peak at minute 21.815 was defined as 3,4-dicaffeoylquinic acid (3,4-DCCA).

Consistent with our investigation, analogous studies have likewise documented comparable constituents within extracts derived from *A. millefolium*. In the HPLC analysis of methanolic extract of *A. millefolium*, 3,4-dicaffeoylquinic acid, 3,5-dicaffeoylquinic acid, and chlorogenic acid were elucidated [19]. In another study, the *A. millefolium* extract contained 7.9% of 3,4-DCCA and 24.2% of 3,5-DCCA [25]. In a study conducted in 2022, 3,5-DCCA was found to be the most abundant phenolic acid at 361.7 mg/100 g. Meanwhile, 3,4-DCCA and chlorogenic acid were present at 38.3 ± 5.1 and 61.7 ± 0.2 mg/100 g, respectively [26]. Lidiya et al. reported that the microwave-assisted extract contained 403.86 μg/g DW of chlorogenic acid, while the decoction extract contained 784.11 μg/g DW [21]. In our study, quantification of the compounds in the extract was determined as chlorogenic acid 27.7 ± 0.90 μg/mL, 3,4-dicaffeoylquinic acid 20.29 ± 0.01 μg/mL, and 3,5-dicaffeoylquinic acid 98.16 ± 2.52 μg/mL.

### 3.5. Formulation studies

#### 3.5.1. Liposome and ethosome formulations: preparation and characterization

**Figure 4:**
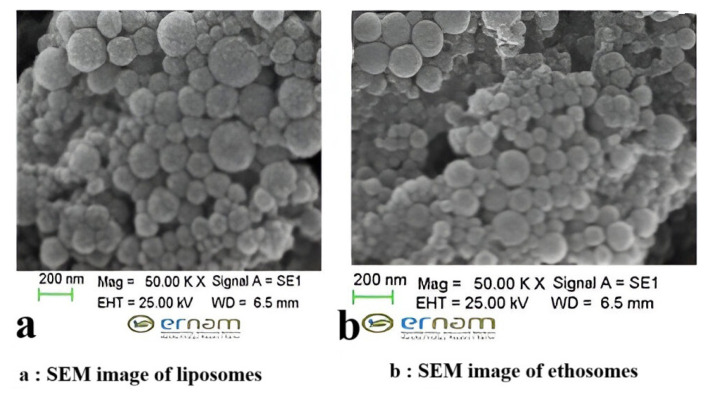
**A**: SEM image of liposomes, **B:** SEM image of ethosomes.

Upon characterization of the ethosomal and liposomal formulations incorporating *A. millefolium* (ACM99.9) extract developed within our investigation, the ethosomal particle size was determined to be 217 ± 1.05 nm, with a zeta potential of 32.5 ± 1.10 mV and a polydispersity index of 0.225 ± 0.013. Furthermore, HPLC analysis showed a release rate of 47.3 ± 1.01% (≈490 μg/mL extract) over 3,4-dicaffeolquinic acid in the release test. The loading efficiency was measured at 52.2 ± 1.40%. Similarly, the liposomal particle size was recorded at 228 ± 1.15 nm, accompanied by a zeta potential of 25.9 ± 1.12 mV and a polydispersity index of 0.230 ± 0.011. The results are given in Table 4. In addition, at liposomal formulation, HPLC analysis showed a release rate of 45.7 ± 1.36% (≈480 μg/mL extract) over 3,4-dicaffeolquinic acid. Upon comparison with the activity assays conducted in the current investigation, it becomes evident that the quantity of extract released from both formulations corresponds to levels that would demonstrate notable efficacy in both antioxidant and cytotoxicity assays. Figures 4A and 4B show vesicular structures intact in the produced ethosomes and liposomes examined by scanning electron microscopy (SEM). Percentage inhibition values of the formulations in cell lines are also shown in Table 4.

Zeta potential is crucial to particle stability and cell adhesion, and particle suspension stability requires a positive or negative zeta potential [27]. Except for levels between −30 and +30 mV, zeta potential values are assumed to drive colloidal stability. Conversely, Van der Waals attraction forces on particles can generate flocculation and particle aggregation at low zeta potential, causing physical instability [28]. The polydispersity index spans from zero, indicating monodisperse particles, to 0.5, signifying broad dispersion. Values surpassing 0.5 impede the achievement of a logarithmic normal distribution of the polydispersity index [29]. These values of our liposome and ethosome formulation are within the desired ranges.

In the investigation conducted by Andleeb et al., *A. millefolium* extract, extracted using 70% ethanol, was utilized to prepare ethosomal formulations [18]. These extract-loaded ethosomes were subsequently incorporated into a topical gel formulation. The ethosomal formulation released 78.6% of the extract, suggesting it might be effective for plant extracts. A stable topical gel with *A. millefolium* penetrates better than standard gels. This suggests that it could be a promising product worthy of additional research. The difference in release rates between this study and ours may be due to ethosomal preparation composition. Hassanzadeh-kiabi and Negahdari examined the antinociceptive effects of aqueous extracts of *A. millefolium* and *Origanum vulgare* L., as well as their liposome-combined extracts, in rats treated with 3% formalin [30]. The maximum antinociceptive effects were 55% for *A. millefolium* and 57% for *O. vulgare*, while the liposome formulations of both extracts produced a 66% effect.

## 4. Conclusions

This study has systematically shown that the solvent composition used at different concentrations significantly affects the phytochemical profile of *A. millefolium* extracts and, consequently, their biological activity. Higher ethanol concentrations (90% and 99.9%) were observed to be more effective in the extraction of total phenolic and flavonoid compounds. The richness of these phytochemicals in the extract was found to be directly related to superior antioxidant capacities, as evidenced by DPPH, ABTS, and FRAP tests. The ACM70 extract was most active against the MDA-MB-231 cell line, while the ACM90 and ACM99.9 extracts showed significant cytotoxic activity against the COLO 205 and HeLa cancer cell lines. Based on this comprehensive bioactivity screening, the extract prepared with 99.9% ethanol was identified as the most promising candidate for further development due to its consistently high activity in both antioxidant and cytotoxicity assays. To enhance the potential of integrating the extract into a formulation, the ACM99.9 extract was loaded into ethosomal and liposomal nanocarriers. The resulting formulations possessed optimal physicochemical properties such as small particle size, low polydispersity, and high zeta potential, indicating good colloidal stability for the carrier systems. In addition, both carrier systems enabled significant release of the extract, with the release amount reaching concentrations within the range shown to be effective for the screened biological activities.

In summary, this study not only shows that the absolute ethanol extract of *A. millefolium* is a rich source of bioactive compounds but also confirms that nanocarrier systems such as ethosomes and liposomes can be used as a promising strategy to improve the formulation and potentially the delivery of this extract and its components for future therapeutic applications.

## Figures and Tables

**Table 1:** Total phenol and flavonoid contents, DPPH and ABTS radical scavenging activity of *A. millefolium* extracts.

Extract	Total phenol [mg_GAE_/g_extract_]	Total flavonoid [mg_CA_/g_extract_]	DPPH IC_50_ (mg/mL)	ABTS TEAC mmol/L Trolox	
				(0.25mg/mL)	(0.5mg/mL)
**ACM10**	83.30 ± 1.60	19.75 ± 3.13	0.39 ± 0.01^5^	0.90 ± 0.04^a^	1.13 ± 0.06^b^
**ACM20**	90.09 ± 0.26	18.29 ± 0.32	0.27 ± 0.04^4^	0.95 ± 0.05^a^	1.09 ± 0.08^b^
**ACM30**	98.26 ± 4.39	21.39 ± 0.52	0.19 ± 0.04^3^	0.98 ± 0.06^a^	1.42 ± 0.11^d^
**ACM40**	110.29 ± 1.87	26.80 ± 0.66	0.11 ± 0.01 ^2^	1.01 ± 0.06^a,b^	1.43 ± 0.09^d^
**ACM50**	115.54 ± 0.53	32.08 ± 1.65	0.07 ± 0.01^1,2^	1.21 ± 0.08^c^	1.78 ± 0.17^f^
**ACM60**	127.27 ± 1.66	44.36 ± 1.05	0.043 ± 0.003^1^	1.45 ± 0.10^d^	2.09 ± 0.16^g^
**ACM70**	143.77 ± 2.13	57.75 ± 0.59	0.037 ± 0.002^1^	1.60 ± 0.12^e^	2.29 ± 0.16^h^
**ACM80**	138.83 ± 5.61	55.30 ± 1.34	0.042 ± 0.003 ^1^	1.49 ± 0.09^d^	2.00 ± 0.12 ^g^
**ACM90**	146.24 ± 0.80	62.73 ± 1.26	0.041 ± 0.001 ^1^	1.38 ± 0.06^d^	2.08 ± 0.09 ^g^
**ACM99.9**	140.23 ± 16.51	58.31 ± 1.31	0.044 ± 0.002 ^1^	1.47 ± 0.10^d^	2.06 ± 0.08 ^g^
**Chlorogenic acid**	-	-	0.05 ± 0.007^1^	0.71 ± 0.04^a^	1.91 ± 0.11^f,g^

Mean ± SD; n = 3. Significant (p < 0.05) differences in each column are shown by different numbers (1–5) and lowercase letters (a–h). ACM10: 10% ethanol extract; ACM20: 20% ethanol extract; ACM30: 30% ethanol extract; ACM40: 40% ethanol extract; ACM50: 50% ethanol extract; ACM60: 60% ethanol extract; ACM70: 70% ethanol extract; ACM80: 80% ethanol extract; ACM90: 90% ethanol extract; ACM99.9: absolute ethanol extract.

**Table 2:** Cytotoxic IC_50_ values of extracts and inhibition% of the formulations in cell lines.

Extracts	IC_50_(μg/mL)		
	COLO	HeLa	MDA-MB-231
**ACM10**	>250	>500	206.23 ± 38.93^e^
**ACM20**	>250	>500	104.75 ± 6.23^c,d^
**ACM30**	>250	>250	136.24 ± 38.37^d^
**ACM40**	237.6 ± 4.23^IV^	>250	56.21 ± 7.52^a,b,c^
**ACM50**	172.9 ± 13.23^III^	164.40 ± 37.88^2,3^	75.46 ± 7.33^b,c^
**ACM60**	153.9 ± 6.23^III^	209.04 ± 44.16^3^	81.78 ± 4.02^b,c^
**ACM70**	103.9 ± 11.23^II^	118.59 ± 24.36^1,2^	30.48 ± 5.15^a^
**ACM80**	101.65 ± 19.97^II^	129.94 ± 12.15^1,2^	45.25 ± 2.95^a,b^
**ACM90**	50.63 ± 3.12^I^	97.01 ± 7.74^1,2^	53.84 ± 1.97^a,b,c^
**ACM99.9**	52.68 ± 2.71^I^	91.46 ± 3.00^1^	44.09 ± 1.41^a,b^
**Inhibition % of formulations**			
**Liposome**	92.59 ± 0.26%	72.21 ± 1.24%	89.58 ± 0.34%
**Ethosome**	93.08 ± 0.13%	73.32 ± 2.35%	89.69 ± 0.39%

* Mean ± SD; n = 3. Significant (p < 0.05) differences in each column are shown by different symbols (I–IV), numbers (1–3) and lowercase letters (a–e). ACM10: 10% ethanol extract; ACM20: 20% ethanol extract; ACM30: 30% ethanol extract; ACM40: 40% ethanol extract; ACM50: 50% ethanol extract; ACM60: 60% ethanol extract; ACM70: 70% ethanol extract; ACM80: 80% ethanol extract; ACM90: 90% ethanol extract; ACM99.9: absolute ethanol extract.

**Table 3:** The obtained results of ACM99.9 extract with HPLC.

Compounds	RT (min)	Calibration equation [y = ax + b]	Correlation coefficient [*r^2^*]	Extract
				ACM99.9 ^a^
**Chlorogenic acid**	6.546	45.978x + 7.70	0.9994	27.7 ± 0.90^b^
**3,4 dicaffeoylquinic acid**	21.815	34.05x – 137.91	0.9996	20.29 ± 0.01
**3,5 dicaffeoylquinic acid**	17.249	46.57x – 69.09	0.9997	98.16 ± 2.52

**Table 4:** Characterization parameters of liposome and ethosome formulations

Sample	Particle size (nm ± SD)	Polydispersity index	Zeta potential (mV ± SD)	Encapsulation efficiency (% ± SD)	Percentage of release in vitro (% ± SD)	Released extract (μg)
**Liposome**	228 ± 1.15	0.230 ± 0.011	25.9 ± 1.12	52.9 ± 1.05	45.7 ± 1.36	483.5
**Ethosome**	217 ± 1.05	0.225 ± 0.013	32.5 ± 1.10	52.2 ± 1.40	47.3 ± 1.01	488.8

* Mean ± SD; n = 3.

## Footnotes

1 World Flora Online Plant List. Asteraceae [online]. Website https://wfoplantlist.org/taxon/wfo-70000001462023-06?page=1[accessed 04 April 2024].

## References

[1] Strzępek-Gomółka M Gaweł-Bęben K Kukula-Koch W *Achillea* species as sources of active phytochemicals for dermatological and cosmetic applications Oxidative Medicine and Cellular Longevity 2021 2021 1 14 10.1155/2021/6643827 PMC8018854 33833853

[2] Candan F Unlu M Tepe B Daferera D Polissiou M Antioxidant and antimicrobial activity of the essential oil and methanol extracts of *Achillea millefolium* subsp. *millefolium* Afan. (Asteraceae) Journal of Ethnopharmacology 2003 87 2 215 220 10.1016/S0378-8741(03)00149-1 12860311

[3] Tuzlacı E Türkiye Bitkileri Geleneksel İlaç Rehberi İstanbul, Türkiye: İstanbul Tıp Kitabevleri; 2016

[4] Ahmadi-Dastgerdi A Ezzatpanah H Asgary S Dokhani S Rahimi E Phytochemical, antioxidant and antimicrobial activity of the essential oil from flowers and leaves of *Achillea millefolium* subsp. *millefolium* Journal of Essential Oil-Bearing Plants 2017 20 2 395 409 10.1080/0972060X.2017.1280419

[5] Enaru B Socaci S Farcas A Socaciu C Danciu C Novel delivery systems of polyphenols and their potential health benefits Pharmaceuticals 2021 14 10 946 10.3390/ph14100946 34681170 PMC8538464

[6] Bawa R Nanopharmaceuticals European Journal of Nanomedicine 2010 3 1 34 40 10.1515/EJNM.2010.3.1.34

[7] Akbarzadeh A Rezaei-sadabady R Davaran S Joo SW Zarghami N Liposome: classification, preparation, and applications Nanoscale Research Letters 2013 8 1 102 10.1186/1556-276X-8-102 23432972 PMC3599573

[8] Touitou E Dayan N Bergelson L Godin B Eliaz M Ethosomes — novel vesicular carriers for enhanced delivery: characterization and skin penetration properties Journal of Controlled Release 2000 65 3 403 418 10.1016/S0168-3659(99)00222-9 10699298

[9] Abd El-Alim SH Kassem AA Basha M Salama A Comparative study of liposomes, ethosomes and transfersomes as carriers for enhancing the transdermal delivery of diflunisal: In vitro and in vivo evaluation International Journal of Pharmaceutics 2019 563 293 303 10.1016/j.ijpharm.2019.04.001 30951860

[10] Zhishen J Mengcheng T Jianming W The determination of flavonoid contents in mulberry and their scavenging effects on superoxide radicals Food Chemistry 1999 64 4 555 559 10.1016/S0308-8146(98)00102-2

[11] Singleton VL Orthofer R Lamuela-Raventós RM Analysis of total phenols and other oxidation substrates and antioxidants by means of folin-ciocalteu reagent Methods in Enzymology 1999 299 152 178 10.1016/S0076-6879(99)99017-1

[12] Hatano T Edamatsu R Hiramatsu M Mori A Fujita Y Effects of the interaction of tannins with co-existing substances. VI.: effects of tannins and related polyphenols on superoxide anion radical, and on 1, 1-Diphenyl-2-picrylhydrazyl radical Chemical and Pharmaceutical Bulletin (Tokyo) 1989 37 8 2016 2021 10.1248/cpb.37.2016

[13] Re R Pellegrini N Proteggente A Pannala A Yang M Antioxidant activity applying an improved ABTS radical cation decolorization assay Free Radical Biology and Medicine 1999 26 9–10 1231 1237 10.1016/S0891-5849(98)00315-3 10381194

[14] Şeker Karatoprak G Göger F Yerer MB Koşar M Chemical composition and biological investigation of *Pelargonium endlicherianum* root extracts Pharmaceutical Biology 2017 55 1 1608 1618 10.1080/13880209.2017.1314511 28407721 PMC7012040

[15] İlgün S Şeker Karatoprak G Evaluation of toxic effects of *Dictamnus albus* L. extracts on PC-12 and SHSY-5Y cell lines and investigation of antioxidant activity Kahramanmaraş Sütçü İmam Üniversitesi Tarım ve Doğa Dergisi 2022 25 Ek Sayi̇ 2 316 325 10.18016/ksutarimdoga.vi.1062822

[16] Yücel Ç Şeker Karatoprak G Development and evaluation of antioxidant effectiveness of chitosan coated liposomes containing caffeic acid Düzce Üniversitesi Sağlık Bilimleri Enstitüsü Dergisi 2021 11 1 8 15 (in Turkish with an abstract in English). 10.33631/duzcesbed.706868

[17] Yücel Ç Karatoprak GŞ Değim İT Antiaging formulation of rosmarinic acid-loaded ethosomes and liposomes Journal of Microencapsulation 2019 36 2 180 191 10.1080/02652048.2019.1617363 31070486

[18] Andleeb M Shoaib Khan HM Daniyal M Development, characterization and stability evaluation of topical gel loaded with ethosomes containing *Achillea millefolium* L. extract Frontiers in Pharmacology 2021 12 1 11 10.3389/fphar.2021.603227 PMC8074965 33912036

[19] Vitalini S Beretta G Iriti M Orsenigo S Basilico N Phenolic compounds from *Achillea millefolium* L. and their bioactivity Acta Biochimica Polonica 2011 58 2 203 209 10.18388/abp.2011_2266 21503279

[20] Keser S Celik S Turkoglu S Yilmaz Ö Antioxidant activity, total phenolic and flavonoid content of water and ethanol extracts from *Achillea millefolium* L Turkish Journal of Pharmaceutical Sciences 2013 10 3 385 392

[21] Lidiya G Gadjalova A Mihaylova D Pavlov A *Achillea millefolium* L.-Phytochemical profile and in vitro antioxidant activity International Food Research Journal 2015 22 1347 1352

[22] Ivanović M Grujić D Cerar J Razboršek MI Topalić-Trivunović L Extraction of bioactive metabolites from *Achillea millefolium* L. with choline chloride based natural deep eutectic solvents: A study of the antioxidant and antimicrobial activity Antioxidants 2022 11 4 724 10.3390/antiox11040724 35453409 PMC9027353

[23] Amirghofran Z Karimi Mh Cytotoxic activity of *Thymus vulgaris*, *Achillea millefolium* and *Thuja orientalis* on different growing cell lines Medical Journal of the Islamic Republic of Iran 2002 15 3 149 154

[24] Pereira JM Peixoto V Teixeira A Sousa D Barros L *Achillea millefolium* L. hydroethanolic extract inhibits growth of human tumor cell lines by interfering with cell cycle and inducing apoptosis Food and Chemical Toxicology 2018 118 635 644 10.1016/j.fct.2018.06.006 29883784

[25] Nateghi N Karimi E Oskoueian E Nanoliposome-encapsulated and nonencapsulated phenolics from *Achillea millefolium* and their biological function in mice challenged by *Campylobacter jejuni*: a comparative study Frontiers in Molecular Biosciences 2022 8 1 8 10.3389/fmolb.2021.832022 PMC8847675 35187077

[26] Villalva M Silvan JM Alarcón-Cavero T Villanueva-Bermejo D Jaime L Antioxidant, antiinflammatory, and antibacterial properties of an *Achillea millefolium* L. extract and its fractions obtained by supercritical antisolvent fractionation against *Helicobacter pylori* Antioxidants 2022 11 10 1849 10.3390/antiox11101849 36290572 PMC9598488

[27] Gaikwad VL Choudhari PB Bhatia NM Bhatia MS Characterization of pharmaceutical nanocarriers: *in vitro* and *in vivo* studies Grumezescu AM Nanomaterials for Drug Delivery and Therapy William Andrew Publishing 2019 33 58 10.1016/B978-0-12-816505-8.00016-3

[28] Joseph E Singhvi G Multifunctional nanocrystals for cancer therapy: a potential nanocarrier Grumezescu AM Nanomaterials for Drug Delivery and Therapy William Andrew Publishing 2019 91 116 10.1016/B978-0-12-816505-8.00007-2

[29] Grau MJ Kayser O Mu RH Nanosuspensions of poorly soluble drugs — reproducibility of small scale production International Journal of Pharmaceutics 2000 196 155 157 10.1016/S0378-5173(99)00411-1 10699708

[30] Hassanzadeh-kiabi F Negahdari B Antinociceptive synergistic interaction between *Achillea millefolium* and *Origanum vulgare* L. extract encapsulated in liposome in rat Artificial Cells, Nanomedicine, and Biotechnology 2018 46 5 994 1000 10.1080/21691401.2017.1354303 28720004

